# What factors delay initiation of bystander CPR in out-of-hospital cardiac arrest? Results from an analysis of 200 recorded ambulance calls

**DOI:** 10.1136/emermed-2024-214733

**Published:** 2026-01-20

**Authors:** Barbara Farquharson, Marie Johnston, Catherine Best, Gareth R Clegg

**Affiliations:** 1 University of Stirling, Stirling, UK; 2 University of Aberdeen, Aberdeen, UK; 3 Medicine, University of Edinburgh, Edinburgh, UK; 4 Emergency Medicine, Royal Infirmary of Edinburgh, Edinburgh, UK

**Keywords:** Cardiopulmonary Resuscitation, Out-of-Hospital Cardiac Arrest, psychology, emergency ambulance systems

## Abstract

**Background:**

Cardiopulmonary resuscitation (CPR) is often not initiated promptly enough in out-of-hospital cardiac arrest, even when call-handlers provide instructions. Identifying the critical, potentially modifiable, barriers to CPR is essential. Our aim was to identify factors associated with delays (1) positioning patient flat and (2) initiating CPR in recordings of cardiac arrest calls and to explore potentially modifiable behavioural factors.

**Methods:**

Retrospective analysis of 200 call recordings to the Scottish Ambulance Service January 2019–December 2020 during which dispatcher-assisted CPR instructions were provided. Potential barriers were coded inductively. Log rank tests were used to explore differences in ‘time to position patient flat’ and ‘time to initiate CPR’ depending on the presence/absence of potential barriers identified.

**Results:**

A random sample of 200 calls were selected from 11 275 potentially eligible calls. Patients in those calls were mostly male (61%), most aged 40–80s; callers were mostly female spouses. Time to position patient flat: median 40 s (IQR: 15.5–82.0), time to initiate CPR: median 50 s (IQR: 36–92). Between 1 and 11 potential barriers were identified in calls (median=4, IQR:2–6).

The most common barriers identified were communication (48%), emotion (45.5%) and physical challenges (38.5%). Various physical challenges, concern patient too heavy, concern that it was too late/futile, concern about physical ability, concern about doing harm and caller being ‘upset’ were significantly associated with delays to positioning the patient flat. Callers ‘not knowing how’ to do CPR; expressing concerns about doing harm, expressing anger and various physical challenges including concerns about ability were associated with delays in initiating CPR. Many significant barriers are potentially amenable to behavioural techniques.

**Conclusion:**

Barriers to ‘positioning the patient flat’ and ‘initiating CPR’ are not the same. Concerns vary, and so identifying and addressing the specific concerns for individual callers at each stage might facilitate earlier CPR. Many of the issues delaying CPR are potentially modifiable with behavioural techniques.

**WHAT IS ALREADY KNOWN ON THIS TOPIC**

- Cardiopulmonary resuscitation (CPR) is often not initiated promptly enough in out-of-hospital cardiac arrest, even when call-handlers provide instructions. To date, CPR has been conceptualised as a single behaviour, and the barriers identified were presumed to apply to the whole endeavour.

**WHAT THIS STUDY ADDS**

- This study has shown that the significant barriers associated with the two separate behaviours of *positioning the patient flat* and *initiating CPR* are not the same, and thus that different interventions may be required at different stages of the call.
- It also shows that many of the most significant barriers (at both stages) may be modifiable using behaviour change techniques, offering a promising avenue for intervention.

**HOW THIS STUDY MIGHT AFFECT RESEARCH, PRACTICE OR POLICY**

- Future research might wish to consider differentiating distinct behaviours within CPR. The feasibility of including behaviour change techniques for call-handlers is a promising avenue to explore.

## Introduction

 Prompt commencement of bystander cardiopulmonary resuscitation (CPR) is the most important factor determining survival from out-of-hospital cardiac arrest (OHCA). Receiving bystander CPR increases survival up to fourfold,^1^ and it is estimated that chances of survival decrease by 10% for every minute without CPR.^2^ Despite the importance of CPR, Shekar *et al*^3^ show that, in most of the world, less than 50% of people experiencing cardiac arrest receive CPR prior to arrival of emergency services.

Dispatcher-assisted CPR (DA-CPR) or telephone/telecommunicator-assisted CPR (T-CPR), where trained call-takers provide real-time instructions to callers about how to perform CPR, increases the provision of CPR^4–6^ and increases survival.^4 7 8^ However, even in settings where DA-CPR is well established, research has shown that up to a third of bystanders do not deliver CPR even when in receipt of instructions over the telephone.^9^ Most recently, Chen *et al*^10^ reported that CPR was delivered in 55%–70% of cases in Taiwan. Time to CPR can vary widely.^11 12^ Improving the quality of the instructions provided by emergency centre personnel (the first link in the chain of survival) is being recognised as critical to increasing rates of CPR and improving survival in OHCA.^13^ Understanding what prevents bystander CPR initiation is key to achieving these improvements.^14^

The barriers to initiation of DA-CPR have been poorly documented, but more recently stronger evidence has begun to emerge.^15–18^ To date, CPR has been conceptualised as a single behaviour, and the barriers identified presumed to apply to the whole endeavour. Taking an approach informed by behavioural science, we consider that there are at least two key behaviours in achieving CPR in OHCA: (1) getting the patient flat on their back on the ground and (2) performing CPR. We hypothesise that the barriers to each of these components will differ.

The aim of this study was to determine factors that delay or impede (1) patient positioning and (2) initiation of CPR in recorded ambulance calls and, in particular, to identify potentially modifiable behavioural barriers at each stage.

## Methods

### Design

A retrospective analysis of 200 emergency call recordings to the Scottish Ambulance Service (SAS) during which call-handlers provided DA-CPR instructions.

### Study setting

The SAS provides an emergency ambulance service to the whole of Scotland (a population of over 5 million people). SAS receives around 700 000 emergency calls per year with around 3100 people having resuscitation attempted after having an OHCA. At the time of data collection, all calls were received by trained call-handlers and managed using the Medical Priority Dispatch System (MPDS), which provides standardised scripts and protocols for medical emergencies, so that appropriate priority levels can be assigned and appropriate instructions (such as DA-CPR for cardiac arrest) provided prior to the arrival of the ambulance crew.

### Participants

We analysed a random sample of calls made to the SAS Emergency Medical Dispatch Centre (EMDC) between 1 January 2019 and 31 December 2020, during which DA-CPR instructions were provided.

#### Inclusion criteria

- Calls where the patient was identified by the SAS emergency call handler’s initial ‘pre-entry’ questions to be not conscious and not breathing normally.
- Calls where CPR instructions were provided by the emergency call handler

#### Exclusion criteria

- Calls where the caller/rescuer was not a layperson (eg, healthcare professional or emergency service personnel).
- Calls where CPR was underway prior to the call-handler giving instruction.
- Calls where the caller was not physically with the patient.
- Calls where the sound quality precluded transcription.
- Calls related to traumatic OHCA, hangings and drownings as these are situations likely to involve factors that make immediate CPR impossible.

### Sampling and sample size

The sample was drawn from the 11 275 calls to EMDC involving CPR between 1 January 2019 and 31 December 2020 (all calls are recorded as digital audio files). A random selection of 200 calls was analysed, a sample size based on previous research.^19^ We estimated this number would yield an estimated 30 calls where CPR was not initiated, a meaningful number to compare with the remainder (n=170). Even among calls where CPR was ultimately performed, barriers might be expected in 50% of calls (ie, n=85), which was considered sufficient to explore barriers and achieve thematic saturation.

### Procedures

Potentially eligible calls were identified by SAS staff using the MPDS classification: calls where the dispatch code assigned by the call-taker indicated cardiac arrest or where CPR or ‘Resus’ appeared in the electronic patient report form between 1 January 2019 and 31 December 2020 (n=11 275). Call ID numbers were entered into SPSS V29. and a random number generator was used to select 200 calls to be retrieved. Retrieved call recordings were placed in a secure drive within the EMDC and accessed only within the EMDC by the research team. Calls were reviewed by BF for exclusion criteria and reasons recorded (see the Results section). Where there were no exclusion criteria, the call was transcribed verbatim by BF or an assistant (see the Acknowledgements section), omitting patient-identifiable information, until transcripts of 200 calls were achieved. All transcripts were checked by a senior member of EMDC auditing team for (1) accuracy and (2) to confirm no personal data, before being uploaded directly to secure storage in the University of Stirling for analysis.

### Analysis

Data were extracted from the call about (1) the age and gender of the person having the cardiac arrest (the ‘patient’). The age of the patient is asked by call-handler as part of the protocol and gender determined based on the pronouns used by the caller, (2) the gender and relationship to the patient of the person who made the call (the ‘caller’). It was sometimes possible to determine the gender of caller from use of pronouns by others at the scene, otherwise estimated based on tone of voice and agreed by two reviewers. The relationship to the patient was recorded if it could be determined from the call.

### Time data from call

The critical time points listed in table 1 were recorded for each call at the time of transcription, so that the time between them could be measured consistently and systematically. We calculated time to first compression (timepoint G–timepoint C) to facilitate comparisons with the previous literature but focused on the two separate behaviours as follows: *Time to position the patient flat* was calculated from end of instruction to position patient flat (timepoint D) and confirmation it was achieved (timepoint E). *Time to initiate CPR* is calculated from end of instruction to perform CPR (timepoint F) to when it was initiated (timepoint G).

**Table 1:** : Time points recorded

Timepoint		Source	Definition
A	Date/time call received	SAS record	Date/time call received as logged on the electronic patient record by SAS
B	Location and contact details confirmed	Call recording	Time location and contact details confirmed by caller
C	Identification of cardiac arrest	Call recording	Time caller confirms patient is both unconscious and not breathing normally
D	Instruction to position patient flat	Call recording	Time call-handler gives first instruction to caller to position patient flat on back on floor
E	Confirmation patient is flat	Call recording	Time caller confirms the patient is flat on their back
F	Instruction to perform CPR	Call recording	Time call-handler gives first instruction to caller to begin CPR
G	Commencement of CPR	Call recording	Time caller indicates CPR has commenced (by themselves/other) OR when compressions can be heard on recording (whichever comes first)

CPR, cardiopulmonary resuscitation; SAS, Scottish Ambulance Service.

### Qualitative data from call

Transcripts were uploaded into QSR Nvivo V.14 software. The researcher (BF) was entirely independent from SAS, with no experience of emergency dispatch but with prior experience of content-analysing medical consultations. The analysis process was as follows:

1. Familiarisation: BF immersed herself in the data (via transcription and reading transcripts) to gain an overall sense of the data.
2. Coding: anything expressed by callers prior to commencing CPR was identified and coded inductively by BF using NVivo.
3. Developing and refining categories: codes were reviewed and grouped into categories of conceptually similar potential barriers (referred to, at this stage, as ‘potential barriers’ as our analysis subsequently showed some to be associated with *faster* CPR behaviour (see below)). Categories were refined through comparison, engagement with the data and refined until each code was coherent and distinctive and a definition created.

Definitions and exemplar quotations are provided in online supplemental file 1. A random sample of 10% transcripts (n=20) were independently coded by a second coder (DD), broadly confirming reliability and the remaining transcripts single-coded. Further reliability checks on a random sample of 20 coded transcripts (with at least one behavioural and psychological barrier) were conducted prior to an intervention development event on 1 June 2023. Each call was independently coded by 2–3 behavioural experts; inter-rater reliability was very high (see online supplemental file 1).

Using SPSS V.28, descriptive statistics were used to summarise patient and caller characteristics. Continuous variables such as the time to event outcomes were summarised as medians and IQRs because of their skewed distributions. Categorical variables such as barriers to CPR were summarised as frequencies and percentages. The relationship between number of barriers and time to event outcomes was assessed using Spearman’s correlation coefficient. Log-rank analysis was run to determine whether there were differences in the distributions of ‘time taken to position patient flat’ and the ‘time taken to initiate CPR’ depending on the presence/absence of main potential barriers identified in the inductive analysis (ie, caller-related identified >5 times). A separate log rank test was run for each barrier as an independent variable with each of the time to event outcomes as dependent variable and no other factors in the analysis. We then conducted Cox regression to test the effect of the presence of the barriers on time to event outcomes after adjusting for gender of the caller.

## Results

Thirty-five of the selected calls were ineligible for the following apriori reasons: CPR is already underway (n=14); traumatic OHCA (n=8); caller remote from the patient (n=3); caller healthcare professional (n=2); OHCA not identified in call (n=4); call terminated (n=2); newborn resuscitation (n=1); sound quality inadequate (n=1) and replaced with further randomly selected calls until sample size of 200 achieved.

### Study population

**Figure 1:**
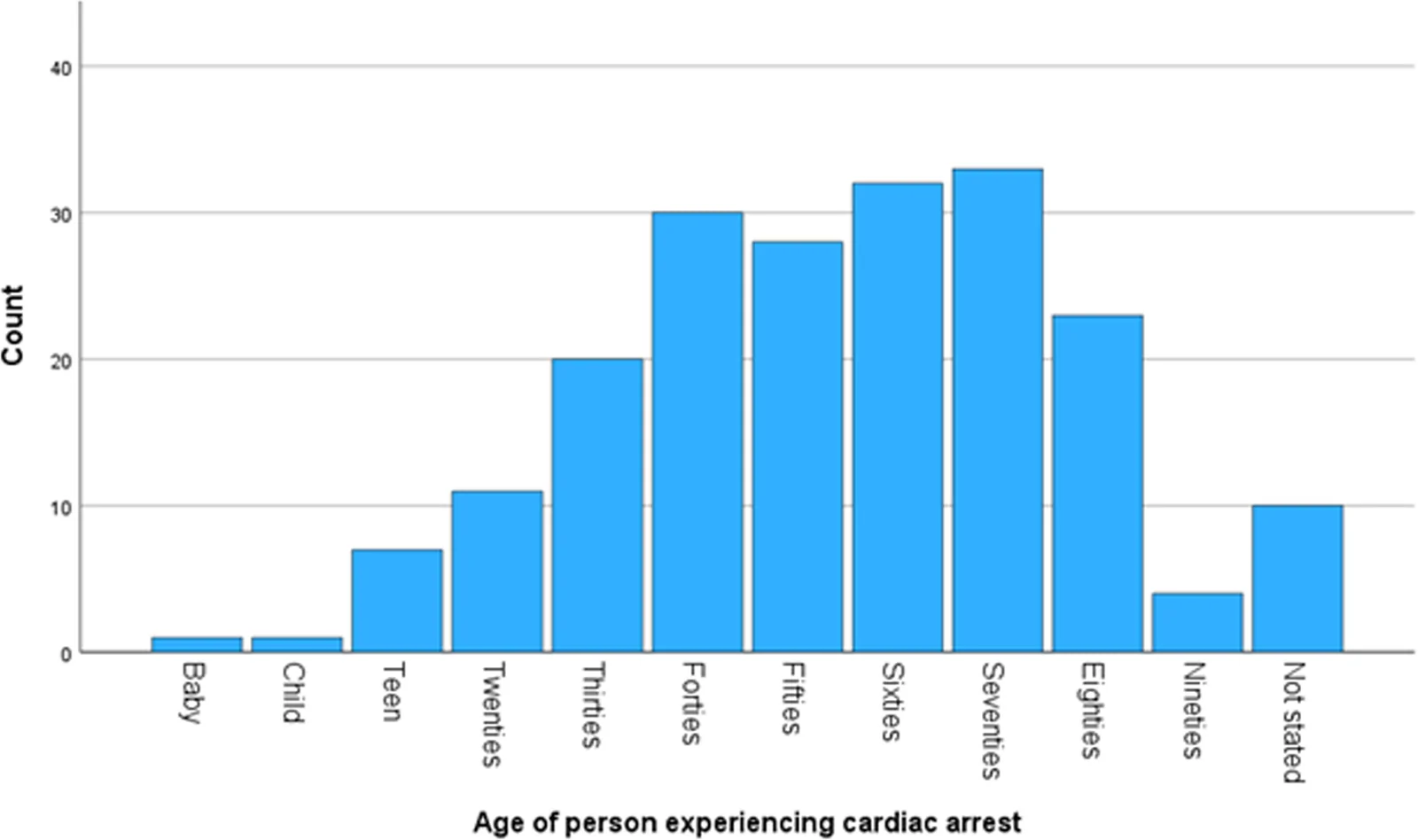
Age of person experiencing cardiac arrest in the 200 analysed calls.

**Figure 2:**
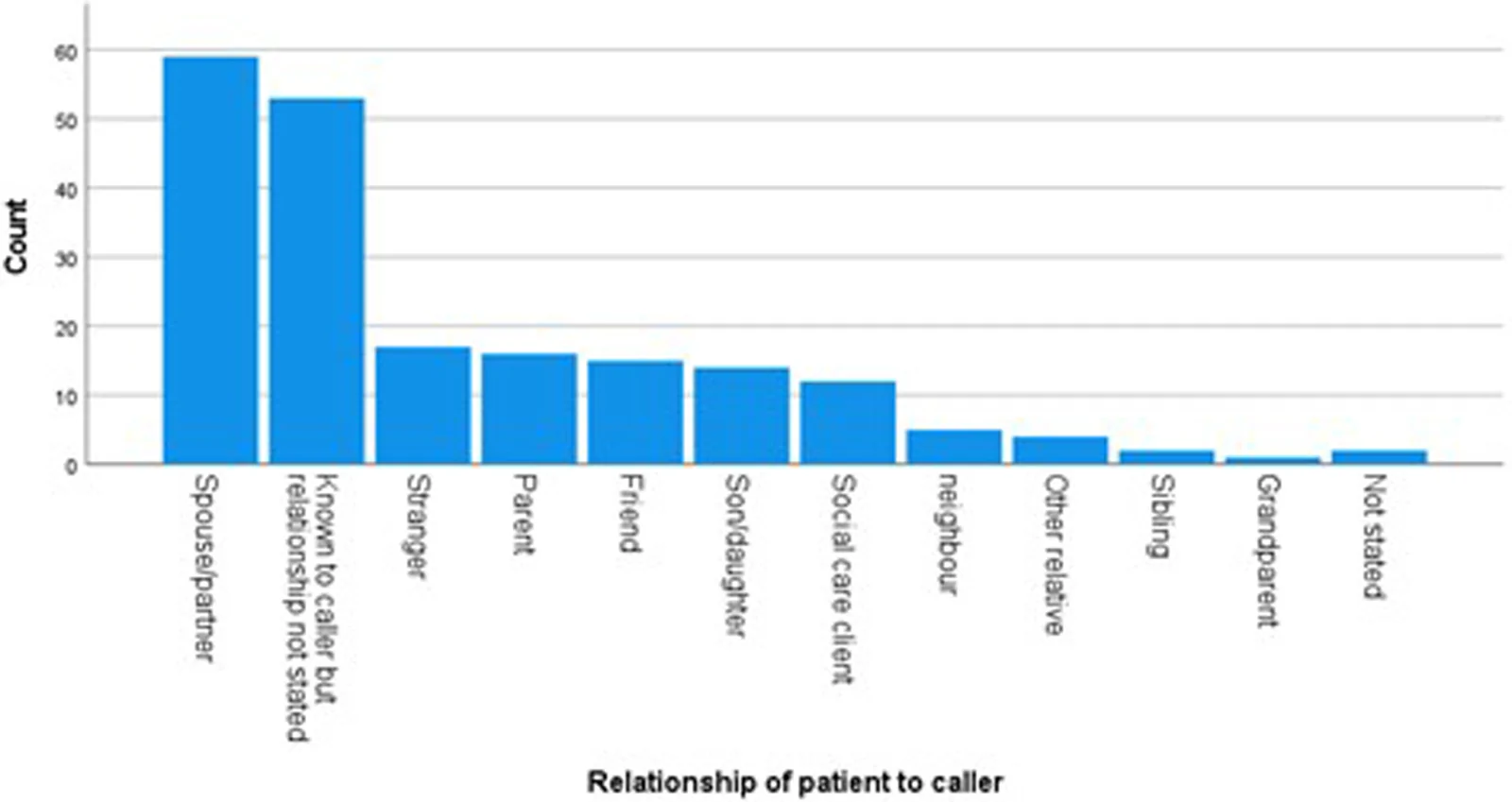
Relationship between patient and caller.

The people experiencing cardiac arrest were male in 122 calls (61%), ages ranged from a baby to over 90 years old, most being in their 40s to 80s (see figure 1). Callers were female in 125 (62.5%) cases, callers were most commonly spouses and were known to patient in the majority of cases (see figure 2), only 17 (8.5%) were strangers.

In 94 (47%) calls, a single person who spoke to the call-handler was identified, in 80 (40%) there were 2, in 16 (8%) 3, in 9 (4.5%) 4 and in 1 case (0.5%) 5 different people spoke during the call.

CPR was achieved in 188 (94%) of calls (in 25 it was audible on call, 87 confirmed by caller, 49 caller counting with effort audible, 14 caller counting, eight assumed by call-handler though never confirmed, five patient responded). In 141 cases (70.5%) CPR was initiated by the person who made the 999 call.

In three cases, the call ended unexpectedly before CPR instructions were provided; in one call, the caller hung up before the instruction was completed; in two calls, CPR started prior to the call-handlers' instruction, generating a negative value for ‘time from instruction to CPR initiation’ and it was not possible to determine whether CPR was initiated in five calls: these 11 calls are therefore ‘missing’ in the Log rank test analysis, below. In three calls where CPR instructions were given: the ambulance arrived before CPR was started in one, the caller resisted before hanging up in another and the patient recovered in another, so data were censored at those points.

**Figure 3:**
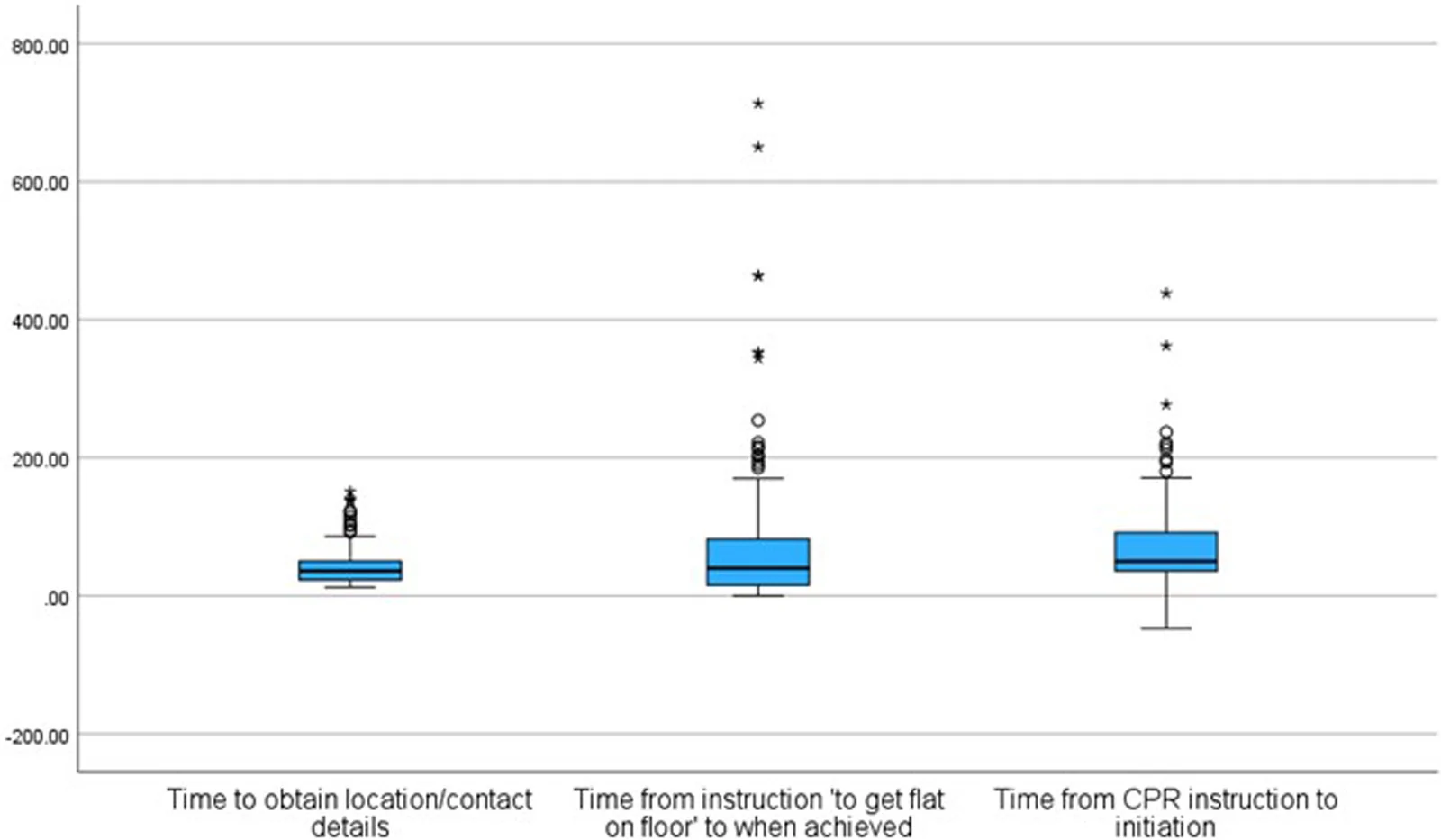
Time intervals. CPR, cardiopulmonary resuscitation.

Time interval data within the calls are summarised in figure 3. Median time to first CPR (from identification of cardiac arrest to first CPR) was 151 s (IQR: 108–223). On average, it took call-handlers a median of 36 s (IQR: 23–50) to obtain the required data for ambulance dispatch (ie, patient location, contact phone number, etc).

The time taken to position the patient flat took a median of 40 s but with much more variation (IQR: 15.5–82.0), the longest time taken to position patient flat being over 11 min. Median time from instruction to CPR initiation was 50 s (IQR: 36–92), the longest interval was over 7 mins.

The main (present in >5/200 calls) potential barriers encountered prior to the initiation of CPR are summarised in online supplemental file 1. Issues with the *identification* of the cardiac arrest are reported in online supplemental file 1 for information but not a focus for analysis in this paper, which is concerned with caller-related behaviours after call-handler identification of cardiac arrest.

The most common potential barriers identified were communication challenges (eg, distorted line, difficulty hearing, having to put the phone down to follow instructions), which were present in 96 (48%) calls; emotion in 91 calls (45%)—anger, panic, shock and most commonly ‘upset’; and challenges physically moving the patient in 77 calls (38.5%). Six potential barriers were identified as potentially amenable to behavioural techniques: emotion (45.5%); concerns about physical capability (33%); concern too late/futile (17.5%); concern about doing harm (8.5%); not knowing how to do CPR (8%); rescuer wanting to do something different from instructions (14%).

Between 1 and 11, separate barriers were identified in calls (median=4, IQR: 2–6). A greater number of barriers was associated with a longer time to position the patient flat (ρ=0.493, p<0.001) and to initiate CPR (ρ=0.245, p<0.001).

### Log rank tests

In table 1, it can be seen that in relation to time taken to get patient flat, callers took longer where there were physical challenges (median time 93.0 s (71.3–114.7) with, 23.0 s (18.0–28.0) without), particularly concerns that patient was ‘too heavy’ (median 136.0 s (104.2–168.8) with, 33.0 s (25.4–40.6) without) or in an awkward position (median 102.0 s (75.7–128.3) with, 27.0 s (22.4–31.6) without); concerns about doing harm (92.0 s (61.1–122.9) with, 36.0 (26.4 to 45.6) without), where caller expressed upset (median 65.0 s (43.7–86.3) with, 34.0 (24.9–43.1) without), expressed concern about ability to physically achieve (median 101.0 s (65.9–136.1) with, 26.0 s (21.5–30.5) without, expressed concern that it might be ‘too late’ or futile (median 66.0 s (26.0–106.0) with, 39.0 s (29.7–48.3) without) but callers were *faster* to achieve positioning patient flat when the caller made efforts to communicate the seriousness of the situation to call-handlers (median 14.0 s (4.0–24.0) with, 45.0 (36.1–53.9) without) and in situations where instructions relating to overdose were provided (median 5.0 s (0–10.1) with, 44.0 (34.1–53.9) without). Results remained significant after controlling for the gender of the caller (Cox regression).

In relation to time taken to initiate CPR, callers took longer where they indicated they ‘didn’t know how’ (median 127.0 s (44.7–209.3) with, 49.0 s (43.8–54.2), expressed concerns about doing harm (median 110.0 s (1.1–218.9) with, 51.0 (45.7–56.23) without, expressed anger (median 100.0 s (196–180.4) with, 50.0 (45.0–55.0) without), concern about ability to physically achieve (median 55.0 s (38.6–71.4) with, 49.0 s (43.1–54.9) without), where there were physical challenges (median 53.0 (39.0–67.1) with, 49.0 (42.2–55.8) without), concerns that patient was ‘too heavy’ (median 66.0 (24.0–108.0) with, 49.0 (44.0–54.0) without) or in an awkward position (median 64.0 (34.4–93.6) with, 47.0 (40.7–53.3) without). For the physical challenges, the median survival times for those with and without barriers are very similar and the CIs for the median overlap. However, the log rank test and HR between groups over the full time period are significantly different. Inspection of the cumulative survival graphs (shown in online supplemental file 2) indicates that the survival distributions diverge after the median survival time, that is, the difference between groups is most apparent in the calls where it takes longer to initiate CPR. Results presented in table 1 remained significant after controlling for the gender of the caller (Cox regression) except *concerns that patient was ‘too heavy’* became non-significant.

## Discussion

This analysis of 200 recorded Scottish ambulance calls identified barriers to DA-CPR and importantly, for the first time, has shown that the barriers impeding patient positioning and CPR initiation itself are not the same. This is important as it suggests that different interventions may be required at different stages of the call to facilitate the required actions.

The most common barriers identified were communication challenges (48%), emotion (45.5%) and physical challenges (38.5%), findings that are highly consistent with recent findings from Aldridge *et al*^18^ and Case *et al*^20^ (both Australia).

Communication challenges were identified by Aldridge *et al*^17^ in 52% of calls and associated with non-initiation of CPR, in 54% by Case *et al*^20^ while Missel *et al*^16^ reported communication gaps in 72% calls (USA), but incorporated what we have separately termed ‘caller clarifying instructions’ into that theme. Communication challenges such as distorted line, difficulty hearing and signal problems appear to be a feature of at least half of OHCA calls.

Emotion was identified as an issue in 45.5% of calls in this study, strikingly similar to the 47% reported by Aldridge *et al*^18^ and consistent with others.^16 21 22^ However, comparisons are not straightforward due to measurement differences (eg, studies where only reasons for non-compliance are counted report lower rates^23–25^) and there may be country-level differences relating to expression of emotion.

Chien *et al*^21^ used the emotional content and cooperation score (ECCS) to differentiate the intensity of emotion and identified that 47% were anxious or moderately upset (ECCS 2–3) and 8% hysterical (ECCS 4–5). Those with ECCS 4–5 were significantly less likely to perform CPR than those with lower scores. In our study, lack of achievement of CPR was rare, but the time taken to achieve it did vary widely and both critical behaviours (patient positioning and initiation of CPR) were significantly slower where there was obvious emotion. Tuffley *et al*^22^ found no significant difference in time to CPR between those who were emotionally stressed and those who were not. Like others,^18 20^ we identified a range of emotions (ie, anger, panic, shock as well as upset/distress), and our results suggest that different emotions may have different impacts—those who were upset were significantly slower to achieve positioning the patient flat and initiation of CPR slower when the caller expressed anger. Studies highlight that emotion often co-occurs with other barriers,^16 18 22^ but the direction of effect has not been determined (ie, whether emotion increases expression of other barriers or whether encountering the barriers increases emotion, both plausible). Clearly, it is challenging to reduce the emotional impact of encountering OHCA, particularly when most callers are related to the patient. However, it is likely to be useful for call-handlers to understand the impact emotion may have at different stages of the call and to have strategies to mitigate the impact of those emotions on behaviour.

We identified physical challenges in 38.5% of calls—this included patients being in awkward positions (eg, wedged between furniture), patients being much heavier than rescuers and caller concerns about their own ability to achieve. Physical challenges have been identified as a barrier to DA-CPR in multiple studies^16 18 21 23 24^ most commonly measured as an inability to move the patient, with reported frequencies ranging from 9%^26^ to 65%.^18^ In the current study, physical barriers and awkward positions were, unsurprisingly, significantly associated with time to position the patient flat (others have noted this barrier often occurs before CPR^18^) and also continued to be significant in relation to actual initiation of CPR.

Among the 66 callers in our analysis who expressed concerns about their ability to physically achieve what was being requested, 34 (52%) nevertheless achieved positioning the patient flat and 50 (75%) performed CPR.

Like others,^16^ we have found that OHCA ambulance calls without barriers to CPR are very rare. Challenges in the context of an unexpected medical emergency are inevitable and some will not be possible to ameliorate. Seven barriers (physical challenges, concern patient too heavy, awkward position, concern it was too late/CPR was futile, concern about ability to physically achieve, concern about doing harm and caller being upset) were significantly associated with delays in positioning the patient flat, independent of the gender of the caller. Six barriers (caller ‘didn’t know how’ to do CPR; expressed concerns about doing harm, being angry, physical challenges, awkward position and concern about ability to physically achieve) were significantly associated with delays in initiating CPR, independent of gender. These are important foci for intervention.

We have shown that apparent ‘reluctance’ to perform CPR can be due to a range of underlying concerns. Means to identify and address specific concerns for individual callers is likely to help facilitate speedier CPR.^16 27^ Caller concerns about it being too late/futile, being able to physically position patient flat/achieve CPR, doing harm, not knowing CPR and encouraging the caller to act despite ‘upset’ are potentially modifiable with established behaviour change techniques (eg, framing/reframing; verbal persuasion about capability^28^) and this is a promising avenue for intervention^29^ in the context of DA-CPR. Our team already has work underway to select behaviour change techniques^30^ likely to be effective in this context and to explore how feasible it is for call-handlers to deliver them—results will be reported in due course and will add to the limited but increasing body of knowledge around how to optimise DA-CPR.^31^

### Strengths and limitations

A particular strength of this study was that calls were selected randomly from across a 2-year period, minimising potential bias. All transcriptions were checked for accuracy by someone external to the study (table 2).

**Table 2:** Relationship of potential barriers with time to position patient flat and initiate CPR

	To get patient flat		To initiate CPR	
Potential barrier	n	HR (95% CI)	n	HR (95% CI)
Defibrillator				
Confusion about defibrillator	193	0.91 (0.61 to 1.36)	189	1.18 (0.79 to 1.77)
Caller relaying instructions to fetch defibrillator	193	1.71 (0.90 to 3.26)	189	1.56 (0.79 to 3.06)
COVID				
Instruction to cover mouth cloth	193	0.98 (0.65 to 1.47)	189	0.70 (0.46 to 1.07)
COVID screening questions	193	0.71 (0.41 to 1.22)	189	0.77 (0.44 to 1.36)
Behavioural				
Don't know how (to do CPR)	193	0.94 (0.56 to 1.57)	189	**0.47 (0.28 to 0.80)**
Concern about doing harm	193	**0.58 (0.35 to 0.96)**	189	**0.48 (0.28 to 0.83)**
Emotion	193	0.78 (0.58 to 1.04)	189	0.91 (0.68 to 1.22)
Anger	193	0.65 (0.35 to 1.24)	189	**0.42 (0.21 to 0.84)**
Panic	193	1.30 (0.86 to 1.97)	189	1.30 (0.85 to 1.98)
Shock	193	0.87 (0.63 to 1.21)	189	0.95 (0.69 to 1.33)
Upset	193	**0.66 (0.48 to 0.90)**	189	0.79 (0.58 to 1.09)
Concern about ability to physically achieve	193	**0.24 (0.17 to 0.34)**	189	**0.68 (0.50 to 0.93)**
Concern too late/futile	193	**0.65 (0.44 to 0.95)**	189	0.77 (0.53 to 1.13)
Trying to communicate seriousness of situation	193	**1.55 (1.07 to 2.25)**	189	1.04 (0.71 to 1.52)
Rescuer wants to do something different from CH instruction	193	0.94 (0.63 to 1.40)	189	0.79 (0.51 to 1.21)
Other				
Caller clarifying instructions	193	1.17 (0.85 to 1.63)	189	0.88 (0.64 to 1.23)
Providing (unrequested) medical information	193	1.06 (0.79 to 1.42)	189	1.19 (0.88 to 1.61)
Situational	193	1.02 (0.60 to 1.72)	189	0.84 (0.49 to 1.42)
Multiple rescuers	193	0.83 (0.57 to 1.19)	189	0.80 (0.54 to 1.17)
Overdose related instructions	193	**2.85 (1.58 to 5.16)**	189	1.16 (0.66 to 2.05)
Communication challenges	193	0.81 (0.61 to 1.08)	189	0.77 (0.58 to 1.03)
Caller not with patient	193	0.73 (0.43 to 1.24)	189	0.75 (0.44 to 1.30)
Physical challenges	193	**0.22 (0.15 to 0.30)**	189	**0.63 (0.47 to 0.86)**
Too heavy	193	**0.35 (0.22 to 0.55)**	189	**0.61 (0.39 to 0.95)**
Awkward position	193	**0.26 (0.18 to 0.36)**	189	**0.57 (0.41 to 0.80)**

Bold p<0.05.

CH: Call Handler

CPR, cardiopulmonary resuscitation.

In common with other studies of this type, there are a number of limitations. Analyses were conducted on historical call recordings and thus there was no opportunity to check interpretations with those involved. However, coding was conducted using NVivo software and so was available for scrutiny. Independent, double-coding of a random selection of call transcripts was undertaken, providing confidence that interpretations were appropriate and reliable.

SAS calls are managed using a well-developed, structured protocol (MPDS) but inevitably do not always follow a predictable sequence, complicating systematic measurement of time intervals within them. We have endeavoured to be explicit about how time periods were measured to enhance replicability (see table 1) but inevitably sometimes judgements had to be made where things were ambiguous (eg, about the exact time of first compression). Double data entry was undertaken and produced the same results. Similarly, where caller gender was not declared explicitly during the call, categorisation was based on the voice of the caller with the potential for incorrect categorisation.

In conclusion, this study has identified key factors that delay or impede (1) patient positioning and (2) initiation of CPR. Callers’ concerns vary and may be different in relation to each behaviour, so identifying and addressing the specific concerns for individual callers at each stage is likely to help facilitate earlier CPR. Many of the barriers to CPR are potentially modifiable with behavioural techniques, offering a promising avenue for intervention. Research exploring how best to equip call-handlers to deliver these techniques and to evaluate effectiveness is urgently required.

## Supplementary material

10.1136/emermed-2024-214733 online supplemental file 1

10.1136/emermed-2024-214733 online supplemental file 2

10.1136/emermed-2024-214733 online supplemental file 3

## Data Availability

Data are available upon reasonable request.
