# Supplementary material for: What factors delay initiation of bystander CPR in out-of-hospital cardiac arrest? Results from an analysis of 200 recorded ambulance calls
Source: Emerg Med J. 2026 Jan 20;43(9):e214733. doi: 10.1136/emermed-2024-214733 (PMC13539858; doi:10.1136/emermed-2024-214733)
Supplement: Supplementary file 1 [file emermed-43-9-s001.docx]

**Improving outcomes of out of hospital cardiac arrest (OHCA): applying behavioural science to enhance telephone assistance and increase rates of cardiopulmonary resuscitation: Stage 1 and 2 protocol**

**Table of contents**

| **Section** | **Page no.** |
| --- | --- |
| Study details | 2 |
| Key study contacts | 2 |
| Study summary | 3 |
| 1. Background and rationale | 4 |
| 2. Aims and Research Questions | 5 |
| 3. Aim 1 Methods   Design  Sample and recruitment  Procedures  Analysis | 6  6  6  6  7 |
| 4. Aim 2 Methods   Design  Sample and recruitment  Procedures  Analysis | 8  8  9  9  9 |
| 5. Ethical Considerations | 10 |
| 6. Publication and dissemination | 10 |
| 7. References | 11 |

**Study details**

**Full title of the study:**

Improving outcomes of out of hospital cardiac arrest (OHCA): applying behavioural science to enhance telephone assistance and increase rates of cardiopulmonary resuscitation.

**Protocol version number and date:**

Version 3.0

**Research reference numbers:**

Sponsor (University of Stirling):

Funder (BHF): FS/20/24/34944

**Key study contacts**

| **Chief Investigator:** | **Dr Barbara Farquharson**  NMAHP-Research Unit, Scion House, Stirling University Innovation Park, University of Stirling  Stirling, FK9 4LA  Email : |
| --- | --- |
| **Collaborators:** | **Dr Gareth Clegg**, University of Edinburgh  **Professor Marie Johnston,** University of Aberdeen |
| **Sponsor representative:** | Karen Murray  Research Enterprise Office  3B1 Cottrell Building  University of Stirling, FK9 4LA  T: 01786 466690 |
| **Funder :** | **British Heart Foundation (BHF)** |

**Study Summary**

| **Study Title:** | Improving outcomes of out of hospital cardiac arrest (OHCA): applying behavioural science to enhance telephone assistance and increase rates of cardiopulmonary resuscitation (CPR). |
| --- | --- |
| **Study Design:** | Mixed Methods study:  **Stage 1:** Theory-informed analysis of telephone-assisted CPR calls  **Stage 2:** A qualitative interview study with ambulance control staff whose role involves providing telephone-CPR (t-CPR) instructions. |
| **Study Participants:** | Stage 1: Anonymous participants in a random sample of calls to the Scottish Ambulance Service (SAS) where CPR instructions were given by control room staff.  Stage 2: People working in UK Ambulance Dispatch Centres whose role involves providing CPR instructions to callers |
| **Planned Size of Sample:** | Stage 1: 200 calls  Stage 2: 30 call-handlers |
| **Follow up duration:** | n/a |
| **Planned Study Period:** | 12 months |
| **Aims** | Aim 1: To examine ambulance service OHCA calls to identify a) bystander barriers to CPR and b) techniques used by call-handlers to facilitate CPR.  Aim 2: To explore call-handlers’ perceptions of what helps people to follow their instructions and how they overcome common barriers that bystanders present. |
| **Research Questions** | 1a) What are the barriers reported by bystanders to undertaking t-CPR?  1b) What techniques do call-handlers employ during calls to facilitate CPR and overcome barriers?  1c) Which, if any, behaviour change techniques (BCTs) do call-handlers use during t-CPR calls?  1d) Are there any techniques (BCTs or otherwise) more common in calls where CPR was initiated quickly by bystander than in calls where CPR was initiated less quickly/not at all.  2a) What are call handlers’ experiences of delivering t-CPR and views about how t-CPR is delivered?  2b) What do call-handlers’ think are the main barriers to CPR and what do they think helps people to initiate CPR and follow t-CPR instructions? |
| **Funding** | British Heart Foundation  Total funding: £289,830 |

# 1. Background and Rationale

*Out-of-hospital cardiac arrest (OHCA) is a serious problem*

Worldwide, OHCA has a devastatingly high mortality rate.^1^ In the UK, survival to discharge is around 10%, whereas other European centres have achieved survival rates of 25%.^2,3,4^ There are approximately 63,500 OHCA/year in Scotland and England.^5^ More than 3000 lives/year could be saved if the best European rates of survival could be matched in the UK^1,4^ and doing so is a strategic priority of all the UK governments and most developed countries.^2,3,6,7^

*Bystander CPR can save lives but is mostly NOT attempted when required*

High quality studies have consistently shown that prompt bystander cardiopulmonary resuscitation (CPR) is the most important factor determining survival from OHCA, increasing survival up to 4-fold.^2,7^ However, only 35%-45% of people trained in CPR actually attempt it when required.^8,9^ Telephone-assisted CPR (t-CPR), where trained call-handlers in the ambulance service provide real-time instructions to callers on how to perform CPR, increases the provision of CPR,^10,11,12^ and increases survival.^13,14,15^ However, even in settings where t-CPR is well established, up to a third of bystanders do not deliver CPR even when in receipt of instructions on the telephone.^16,17,18,19^ Thus, many people are missing out on life-saving CPR because bystanders do not follow call-handler instructions. If the proportion of people who follow call-handler instructions could be increased, lives would be saved. A recent systematic review has highlighted that there is very little evidence (only 10 studies) about how to improve the effectiveness of t-CPR instructions^20^: giving continuous verbal support, simplified instructions (e.g. compression-only^21^, practical changes about positioning^22,23^ and clothing^24^ were beneficial whereas asking people to put the phone down^25^ and information about compression depth were not^26^.

*Behavioural science can help*

Performing CPR is behaviour but behavioural science has tended not to be systematically applied to CPR. Behavioural science provides a body of evidence about how to change behaviour. It can contribute by **specifying the determinants of behaviour and therefore what to target in order to** **change CPR behaviour.** Identifying barriers to behaviour, using behavioural science to understand those barriers and selecting behaviour change techniques with the potential to address them, has been used to change a variety of behaviours^27,28,29^ and so we expect bystander CPR behaviour will be similarly amenable to change. This protocol specifies systematic development work to inform the development of behaviourally-informed t-CPR instructions which will then be tested in a simulation trial.

**Aims:**

Aim 1: To examine ambulance service OHCA calls to identify a) barriers to CPR and b) techniques used by call-handlers to facilitate CPR.

Aim 2: To explore call-handlers’ perceptions of what helps people to follow their instructions and how they overcome common barriers they encounter.

# 2. Research questions

**Aim 1**

1a) What are the barriers reported by bystanders to undertaking t-CPR?

1b) What techniques do call-handlers employ during calls to facilitate CPR and overcome barriers?

1c) Which, if any, behaviour change techniques (BCTs) do call-handlers use during t-CPR calls?

1d) Are there any techniques (BCTs or otherwise) more common in calls where CPR was initiated quickly by bystander than in calls where CPR was initiated less quickly/not at all?

**Aim 2**

2a) What are call-handlers’ experiences of delivering t-CPR and views about how t-CPR is delivered?

2b) What do call-handlers’ think are the main barriers to CPR and what do they think helps people to initiate CPR and follow t-CPR instructions?

# 3. Methods

# Aim 1

**Design**

A theory-informed content analysis of telephone-CPR calls.

**Study setting**

The Scottish Ambulance Service which provides an emergency ambulance service to the whole of Scotland (a population of over 5 million people).

**Participants**

Anonymous participants in a random sample of calls to the Scottish Ambulance Service (SAS) Emergency Medical Dispatch Centre (EMDC) where t-CPR instructions were provided.

*Inclusion criteria*

- Calls where the patient is ascertained to be not conscious and not breathing normally

*Exclusion criteria*

- calls where the caller/rescuer is not a lay-person (e.g. health care professional or emergency service personnel)
- calls where CPR is underway prior to the call-handler giving instruction
- calls where the caller is not physically with the patient
- calls where the sound quality precludes transcription
- calls relating to traumatic OHCA, hangings and drownings (situations likely to involve factors that make immediate CPR impossible)

**Sampling and sample size**

The sample will be drawn from all calls to EMDC involving CPR in the prior 12 month period. All emergency calls taken by EMDC are recorded as digital audio files. A random selection of 200 calls will be analysed. Existing evidence suggests around 15% of calls where CPR instructions are given by ambulance staff do not result in initiation of CPR^16^ so a sample of 200 is expected to yield an estimated 30 calls where CPR is not initiated, a meaningful number to compare with the remainder (n=170). Even amongst the calls where CPR was ultimately performed, barriers might be expected in around 50% (i.e n=85) and thus this is considered a sufficient number in which to explore barriers and achieve theoretical and thematic saturation.

**Procedures**

Potentially eligible calls involving CPR in the prior 12 month period will be identified by SAS staff using the Medical Priority Dispatch System (MPDS)™ classification: calls will be where the dispatch code assigned by the call-taker indicates cardiac arrest or where CPR or ‘RESUS’ appear in the electronic patient report form will be identified. The total number of calls identified will be recorded and randomly sorted using random number generation in SPSS. The first 200 calls identified will be placed in a secure drive in an Ambulance Control Centre drive made available for the project. Calls will be accessed in the randomised order by either an ambulance service call-handler or BF* who will initially listen to the call to screen for exclusion criteria. Reasons for each exclusion will be recorded. If there are no exclusion criteria the call will be transcribed verbatim, date/time stamping each time the participants in the call speak but omitting ALL patient-identifiable information until the desired sample size of around 200 eligible calls is achieved. Anonymised transcripts will be stored within the SAS ACC drive until it has been confirmed by a senior member of ACC that the transcripts are a) accurate and b) do not contain personal data. They will then be shared with the research team for analysis by uploading directly to a secure Sharepoint folder in the University of Stirling.

*It was originally planned that a call-handler from SAS would transcribe the calls for the research. However, due to the COVID-19 pandemic the SAS is extremely stretched and cannot guarantee availability of a call-handler to do this and so seeking permission for BF to access calls to transcribe as an alternative.

**Analysis**

*Time data from call*

The following critical time-points are defined below and will be recorded for each call so that the time between them can be measured consistently and systematically and calls with prompt initiation of CPR compared with calls with slower/no initiation.

| ***Timepoint*** | ***Source*** | ***Definition*** |
| --- | --- | --- |
| Time call received | SAS record | Time call received as logged by SAS |
| Identification of cardiac arrest | Transcript | Time caller confirms patient is both unconscious and not breathing normally |
| Estimated time of cardiac arrest (if available) | Transcript | Time arrest occurred – either from caller report (e.g. reported time, few minutes ago or unknown) ***OR*** exact time if occurs during call |
| Instruction to perform CPR | Transcript | Time call-handler gives first instruction to caller to begin CPR |
| Commencement of CPR | Transcript | Time caller indicates CPR has commenced (by themselves/other will be noted) ***OR*** when compressions can be heard on recording (whichever comes first) |
| Defibrillation (if occurs) | Transcript | Time of defib arrival  Time first shock is delivered |
| Change of rescuer (if occurs) | Transcript | Time caller indicates someone else has taken over CPR (or is heard on call) |
| End of lay CPR | Transcript | Time bystander CPR attempt ends – it will be noted if this is because   - emergency staff take over - rescuer becomes exhausted - patient recovers - other |

*Qualitative data from call*

Anything expressed as a barrier to commencing CPR will be identified and coded in transcripts, classified as being expressed prior to or after call-handler instruction. Procedural barriers (e.g. language/communication difficulties), technological barriers and physical barriers will be identified, as in previous work^30^ In addition, particular attention will be paid to identifying psychological or behavioural barriers and facilitators - these will be coded using the Theoretical Domains Framework, a validated integrated framework of behavioural theory ^31^.

Call handler responses to presented barriers/resistance will be identified and coded in transcripts and categorised as either 1) successful (caller overcomes barrier and performs CPR), 2) partially successful (caller attempts what handler has instructed but doesn’t work [eg attempts to roll patient onto back] OR caller overcomes barrier but encounters another or 3) not successful (barrier is not overcome, CPR is not performed).

Call-handler instructions will be independently coded for the presence of BCTs by two trained coders (BF and MJ) using the definitions in the BCT Taxonomy V1.^32^ Level of agreement will be reported and any disagreements resolved by a third coder (DD).

Finally, inductive thematic analysis of call-handler instructions other than those coded as BCTs will be conducted.

Descriptive statistics will be used to explore whether particular techniques are more common in calls where CPR is achieved promptly than others.

# Aim 2

**Design**

A qualitative interview study of ambulance staff with experience of providing t-CPR instructions.

**Study setting**

UK Emergency Dispatch Centres

**Participants**

A purposive sample of people working in UK Emergency Dispatch Centres whose role involves providing CPR instructions to callers will be interviewed, aiming for diversity in terms of years of experience, age, gender, geographical location and self-reported confidence about t-CPR calls.

*Inclusion criteria*

- people working in UK Emergency Dispatch Centres whose role involves providing CPR instructions to callers

*Exclusion criteria*

- none

**Sampling and sample size**

Call handlers working in UK EMDCs are the population of interest. We will seek a sample of approximately seven EMDCs – a number adequate to provide diversity in terms of geographical location, urban/rural, size (population served), dispatch software used (MPDS and Pathways), published outcomes for Return of Spontaneous Circulation in OHCA pre-pandemic (e.g. <https://www.england.nhs.uk/statistics/wp-content/uploads/sites/2/2020/12/AmbCO-for-2019-Dec.xlsx>) and latest Care Quality Commission Rating. A purposive sample of approximately 30 call-handlers that is diverse in terms of, age, gender, years of experience and confidence in providing CPR instructions will be sampled from the seven EMDCs.

**Procedures**

Eligible potential participants will be identified by the employing organisation. Potential participants will be approached by an email to their work address (see Appendix 1, Invitation Letter), providing them with information about the study (see Appendix 2, Participant Information Sheet) and inviting them to take part. Information about the study may also be placed in staff bulletins, inviting staff who are interested to contact the researcher. Those interested in taking part will be asked to contact the researcher by telephone or email and given the opportunity to ask questions. Those who wish to take part will be asked to complete and sign a consent form (see Appendix 3). Data collection will only take place after written consent has been obtained.

Only if required to obtain the desired sample (number and diversity), a reminder letter and PIS will be sent to eligible staff members who have not responded. This will be sent no sooner than 2 weeks after the initial letter of invitation.

Semi-structured qualitative interviews will be conducted with consenting call-handlers (see Interview Schedule, Appendix 4). We will explore call-handlers’ experiences of providing CPR instructions to callers, their perceptions of the most common barriers and the techniques they use to help people initiate CPR.

Interviews with participants will be conducted in person where possible. If this is not desired by the participant or not possible (e.g. due to COVID 19 restrictions) interviews will be conducted via video-conferencing / telephone instead. Interviews will be audio-recorded using an encrypted digital voice recorder (in person or telephone interviews). Recorded interviews will be transcribed verbatim, anonymised and entered into NVivo v12 for content coding and thematic analysis. Participants will receive a £50 voucher payment to compensate them for their time.

**Analysis**

Data will be anonymised and initially content coded, referring to the interview schedule for a priori issues and highlighting emergent topics. Transcripts will be second coded by a second researcher to ensure rigour and reliability. Analytic summaries will focus on participant views and experiences and techniques used to aid initiation of CPR by bystanders. A constant comparison method and framework analysis will be used to develop a deep and nuanced account of call-handlers’ perspectives of t-CPR and to summarise their collective tacit knowledge. The interviews will also provide important information about the context in which the intervention will be implemented.

# 6. Ethical Considerations

## Risks to transcribers

Many of the calls being transcribed will be distressing calls to listen to. For this reason, experienced call handlers will be selected to undertake this work. They will be encouraged to undertake the work in short sessions and to let the research team/ line-manager know if they are experiencing distress. To promote a consistent approach it is anticipated that a sole call-handler will transcribe the calls but if difficulties are encountered additional call-handlers will be trained. The support mechanisms provided to SAS call-handlers will be available to those undertaking the transcription work, as well as the support of the research team who will actively check on transcribers weekly. BF is an experienced nurse and has prior experience of transcribing ‘real-time’ calls (although not as distressing as these might be), she will follow all the above procedures and seek support from supervisors if she is finding it unduly distressing.

**Potential identification of poor practice**

It is not the purpose of the research to assess the quality of practice in telephone CPR instructions and consent has not been obtained from call-handlers to do so. Call-handlers follow stringent MPDS protocols and their calls are regularly audited to assess adherence and address performance issues if necessary. These mechanisms are considered adequate to protect the public and therefore the research team will not take any action in relation to practice observed.

## Confidentiality & Data Protection

It is essential that the personal data of those who have been in contact with health services remains confidential ~~and is not disclosed to the research team~~. All data (call recordings and patient data) will remain within the secure environment of the SAS. Personal data will be accessed by the call-handlers (they will hear people’s names/phone numbers etc. on calls as they do routinely in the course of their work) but these details will not be recorded for the purposes of this research. Access for BF will be provided by the SAS to copies of the identified, potentially eligible calls on an ACC drive within the control room with no facility to download or share. Secure login details will be provided to BF to allow her to access the data. She will hear personal information during these calls (e.g. names and addresses) but will transcribe OMITTING all personal information. The anonymised transcriptions will be stored in the ACC drive until they have been checked by ACC staff for a) accuracy and b) to ensure they do not contain any personal data before being shared with the University of Stirling using secure data transfer process (i.e. uploading directly to secure Sharepoint file) for analysis. BF is a Registered Nurse and bound by NMC Professional standards, including to ‘Respect people’s right to privacy and confidentiality’ at all times. https://www.nmc.org.uk/standards/code/read-the-code-online/

~~Calls will be transcribed omitting ALL personal information and then shared with the research team.~~ Each transcription will be stored using a unique identifier which will be connected to a SAS call ID to facilitate accuracy checks but this link document will only be available to the research team and would not allow anyone without clinical access to SAS systems to access that information.

All transcriptions (including calls and call-handler interviews) will be stored in a secure folder on a University of Stirling computer, which is password protected and encrypted. After study completion, research data will be stored for 10 years in DataSTORRE the University Of Stirling online digital repository, after this time data will be securely destroyed. Data retention and destruction will be handled in line with the University of Stirling data management policy.

# 7. Publication and Dissemination

It is anticipated that results will be written up in a report for the funder and published in relevant peer-reviewed journals (e.g. Resuscitation). We will also write an article for a high-quality journalistic style publication such as 'The Conversation' and use social-media tools such as Twitter to facilitate dissemination to the larger 'lay' and clinical population. We will host a dissemination workshop at the end of the project to discuss results and next steps with key stakeholders identified throughout the project and any other interested parties.

# 8. References

1. Berdowski J, Berg RA, Tijssen JG, Koster RW. Global incidences of out-of-hospital cardiac arrest and survival rates: systematic review of 67 prospective studies. Resuscitation. 2010;81(11):1479-87.
2. Scottish Government. Out-of-hospital cardiac arrest: A Strategy for Scotland. Scottish Government 2015.
3. British Heart Foundation. Consensus paper on out-of-hospital cardiac arrest in England.

<https://www.resus.org.uk/publications/consensus-paper-on-out-of-hospital-cardiac-arrest->in-england/; 2014. Contract No.:17/01/2018.

4. Lindner TW, Søreide E, Nilsen OB, Torunn MW, Lossius HM. Good outcome in every fourth resuscitation attempt is achievable—an Utstein template report from the Stavanger region. Resuscitation. 2011;82(12):1508-13.
5. Government S. Scottish Out-of-Hospital Cardiac Arrest data linkage project: initial results Edinburgh: Scottish Government; 2017 [Available from: <https://www.gov.scot/publications/initial-results-scottish-out-hospital-cardiac-arrestdata-linkage-project/>.
6. Gräsner J-T, Lefering R, Koster RW, Masterson S, Böttiger BW, Herlitz J, et al. EuReCa ONE⿿27 Nations, ONE Europe, ONE Registry: A prospective one month analysis of out-of-hospital cardiac arrest outcomes in 27 countries in Europe. Resuscitation. 2016;105:188-95.
7. Graham R, McCoy MA, Schultz AM. Strategies to improve cardiac arrest survival: a time to act: National Academies Press Washington (DC):; 2015.
8. Hawkes C, Booth S, Ji C, Brace-McDonnell SJ, Whittington A, Mapstone J, Cooke MW, Deakin CD, Gale CP, Fothergill R, Nolan JP, Rees N, Soar J, Siriwardena AN, Brown TP, Perkins GD; OHCAO collaborators. Epidemiology and outcomes from out-of-hospital cardiac arrests in England. Resuscitation. 2017 Jan;110:133-140. doi: 10.1016/j.resuscitation.2016.10.030. Epub 2016 Nov 17. PMID: 27865775.
9. Swor R, Khan I, Domeier R, Honeycutt L, Chu K, Compton S. CPR training and CPR performance: do CPR‐trained bystanders perform CPR? Academic Emergency Medicine. 2006;13(6):596-601.
10. Bobrow BJ, Spaite DW, Vadeboncoeur TF, Hu C, Mullins T, Tormala W, et al. Implementation of a Regional Telephone Cardiopulmonary Resuscitation Program and Outcomes After Out-of-Hospital Cardiac Arrest. JAMA Cardiology.2016;1(3):294-302.
11. Harjanto S, Na MX, Hao Y, Ng YY, Doctor N, Goh ES, et al. A before-after interventional trial of dispatcher-assisted cardio-pulmonary resuscitation for out-of-hospital cardiac arrests in Singapore. Resuscitation. 2016;102:85-93.
12. Tanaka Y, Taniguchi J, Wato Y, Yoshida Y, Inaba H. The continuous quality improvement project for telephone assisted instruction of cardiopulmonary resuscitation increased the incidence of bystander CPR and improved the outcomes of out-of-hospital cardiac arrests. Resuscitation. 2012;83(10):1235-41.
13. Wu C-H, Shih C-L, Huang S-C, Ho S-A, Liu Y-P, Lee B-C, et al. Outcomes comparison between a community-wide bystander defibrillation program and dispatcher-assisted CPR in out-of-hospital cardiac arrest at public locations. Resuscitation. 2017;118:e39.
14. Akter G, Hasan Z, Sana D, Nayem S, Islam M. The Impact of Dispatcher Assistance in the Rates and Efficacy of Bystander CPR: A Meta-analysis. Emerg Med (Los Angel). 2017;7(359):2.
15. Nikolaou N, Dainty KN, Couper K, Morley P, Tijssen J, Vaillancourt C, et al. A systematic review and meta-analysis of the effect of dispatcher-assisted CPR on outcomes from sudden cardiac arrest in adults and children. Resuscitation. 2019;138:82-105.
16. Oman G, Bury G. Use of telephone CPR advice in Ireland: Uptake by callers and delays in the assessment process. Resuscitation. 2016 May 1;102:6-10.
17. Maier M, Luger M, Baubin M. Telephone-assisted CPR. Notfall+ Rettungsmedizin. 2016;19(6):468-72.
18. Rea TD, Eisenberg MS, Culley LL, Becker L. Dispatcher-assisted cardiopulmonary resuscitation and survival in cardiac arrest. Circulation. 2001;104(21):2513-6.
19. Riou M, Ball S, Whiteside A, Bray J, Perkins GD, Smith K, et al. ‘We’re going to do CPR’: A linguistic study of the words used to initiate dispatcher-assisted CPR and their association with caller agreement. Resuscitation. 2018;133:95-100.
20. Chen K-Y, Ko Y-C, Hsieh M-J, Chiang W-C, Ma MH-M. Interventions to improve the quality of bystander cardiopulmonary resuscitation: A systematic review. PloS one. 2019;14(2):e0211792.
21. Birkenes TS, Myklebust H, Neset A, Kramer-Johansen J. Quality of CPR performed by trained bystanders with optimized pre-arrival instructions. Resuscitation. 2014;85(1):124-30.
22. Dias J, Brown T, Saini D, Shah R, Cofield S, Waterbor J, et al. Simplified dispatch-assisted CPR instructions outperform standard protocol. Resuscitation. 2007;72(1):108-14.
23. Rasmussen SE, Nebsbjerg MA, Krogh LQ, Bjørnshave K, Krogh K, Povlsen JA, et al. A novel protocol for dispatcher assisted CPR improves CPR quality and motivation among rescuers—A randomized controlled simulation study. Resuscitation. 2017;110:74-80.
24. Chavez DE, Meischke H, Painter I, Rea TD. Should dispatchers instruct lay bystanders to undress patients before performing CPR? A randomized simulation study. Resuscitation. 2013;84(7):979-81.
25. Brown TB, Saini D, Pepper T, Mirza M, Nandigam HK, Kaza N, et al. Instructions to “put the phone down” do not improve the quality of bystander initiated dispatcher-assisted cardiopulmonary resuscitation. Resuscitation. 2008;76(2):249-55
26. Van Tulder R, Roth D, Krammel M, Laggner R, Heidinger B, Kienbacher C, et al. Effects of repetitive or intensified instructions in telephone assisted, bystander cardiopulmonary resuscitation: an investigator-blinded, 4-armed, randomized, factorial simulation trial. Resuscitation. 2014;85(1):112-8.
27. Campbell NC, Murray E, Darbyshire J, Emery J, Farmer A, Griffiths F, et al. Designing and evaluating complex interventions to improve health care. BMJ : British Medical Journal. 2007;334(7591):455-9.
28. Smits S, McCutchan G, Wood F, Edwards A, Lewis I, Robling M, et al. Development of a Behavior Change Intervention to Encourage Timely Cancer Symptom Presentation Among People Living in Deprived Communities Using the Behavior Change Wheel. Annals of behavioral medicine : a publication of the Society of Behavioral Medicine. 2018;52(6):474-88.
29. Debono D, Taylor N, Lipworth W, Greenfield D, Travaglia J, Black D, et al. Applying the Theoretical Domains Framework to identify barriers and targeted interventions to enhance nurses’ use of electronic medication management systems in two Australian hospitals. Implementation Science. 2017;12(1):42.
30. Bray J, Case R, Cartledge S, Siedenburg J, Smith K, Barger B, Finn J. Barriers to bystander cardiopulmonary resuscitation (CPR) in a dispatcher CPR system: a qualitative review of emergency calls. Resuscitation. 2017 Sep 1;118:e90.
31. Cane, J., O’Connor, D. & Michie, S. Validation of the theoretical domains framework for use in behaviour change and implementation research. Implementation Sci 7, 37 (2012). https://doi.org/10.1186/1748-5908-7-37
32. Michie S, Richardson M, Johnston M, Abraham C, Francis J, Hardeman W, et al. The behavior change technique taxonomy (v1) of 93 hierarchically clustered techniques: building an international consensus for the reporting of behaviour change interventions. Annals of behavioral medicine : a publication of the Society of Behavioral Medicine. 2013;46.

Appendix 1: Invitation Letter

Appendix 2: Participant Information Sheet

Appendix 3: Consent form

Appendix 4: Interview Schedule
