# Supplementary material for: What factors delay initiation of bystander CPR in out-of-hospital cardiac arrest? Results from an analysis of 200 recorded ambulance calls
Source: Emerg Med J. 2026 Jan 20;43(9):e214733. doi: 10.1136/emermed-2024-214733 (PMC13539858; doi:10.1136/emermed-2024-214733)
Supplement: Supplementary file 2 [file emermed-43-9-s002.docx]

Supplementary material: Log Rank curves

The survival function estimates the probability that the behaviour has not yet occurred at a particular timepoint. The cumulative survival starts at 1.0 (100%) and goes down over time as the behaviour occurs.

On both curves, censoring of cases where the behaviour definitely did not occur during the call) is marked with a cross.

# Time to position patient flat

Median with 48s, without 40s. Log rank test ns

Median time with 30s without 41s Log rank ns

Median time with 43s, without 40s Log rank test ns

Median time with 68s, without 38s Log rank test ns

Median time with 36s without 41s Log rank ns

Median time 92.0s (61.1-122.9) with, 36.0 (26.4-45.6) without Log rank chi square 4.696 p=0.030

Median time 54s with and 34s s without Log rank ns

Median with 73s and without 39s Log rank test ns

Median time with 31s and without 43s Log rank test ns

Median time with 36s and without 41s. Log rank ns

Median time with 65s and without 34s. Log rank chi square 6.949 p=0.008

Median time with 101s and without 26s. Log rank chi square 72.315 p<0.001

Median time with 66s and without 39s. Log rank chi square 5.051 p=0.025

Median time with 14s and without 45s. Log rank chi square 5.414 p=0.020

Median time with 40s and without 41s. Log rank ns

Median time with 41s and without 39s. Log rank ns

Median time with 39s and without 41s. Log rank ns

Median time with 41s and without 39s. Log rank ns

Median time with 51s and without 39s. Log rank ns

Median time with 5s and without 44s. Log rank chi square 13.340 p<0.001

Median time with 51s and without 32s. Log rank ns

Median time with 39s and not with 57s. Log rank ns

Median time with 93s and without 23s. Log rank chi sq 90.948 p<0.001

Median time with 136s and without 33s. Log rank chi sq 22.410 p<0.001

Median time with 102s and without 27s. Log rank chi sq 67.311 p<0.001

# CPR initiation

Median time with 51s and without 51s. Log rank ns

Median time with 51s and without 51s. Log rank ns

Median time with 60s and without 49s. Log rank ns

Median time with 87s and without 50s. Log rank ns

Median time with 127s and without 49s. Log rank chi sq 8.216 p=0.004

Median time with 110s and without 51s. Log rank chi sq 6.557 p=0.010

Median time with 52s and without 49s. Log rank ns

Median time with 100s and without 50s. Log rank chi sq 6.522 p=0.011

Median time with 41s and without 52s. Log rank ns

Median time with 49s and without 50s. Log rank ns

Median time with 53s and without 50s. Log rank ns

Median time with 55s and without 49s. Log rank chi sq 6.072 p=0.014

Median time with 52s and without 52s. Log rank ns

Median time with 51s and without 51s. Log rank ns

Median time with 51s and without 51s. Log rank ns

Median time with 58s and without 47s. Log rank ns

Median time with 51s and without 51s. Log rank ns

Median time with 46s and without 51s. Log rank ns

Median time with 58s and without 49s. Log rank ns

Median time with 50s and without 51s. Log rank ns

Median time with 54s and without 46s. Log rank ns

Median time with 50s and not with 66s. Log rank ns

Median time with 53s and without 49s. Log rank chi sq 8.766 p=0.003

Median time with 66s and without 49s. Log rank chi sq 5.030 p=0.025

Median time with 64s and without 47s. Log rank chi sq 11.555 p<0.001
