# Supplementary material for: What factors delay initiation of bystander CPR in out-of-hospital cardiac arrest? Results from an analysis of 200 recorded ambulance calls
Source: Emerg Med J. 2026 Jan 20;43(9):e214733. doi: 10.1136/emermed-2024-214733 (PMC13539858; doi:10.1136/emermed-2024-214733)
Supplement: Supplementary file 3 [file emermed-43-9-s003.docx]

| **Potential barriers identified** | **Present in  (n) calls** | **%** | **Definition** | **Example** | **10% reliability  check 1 (kappa)** | **10% reliability  check 2 (kappa)** |
| --- | --- | --- | --- | --- | --- | --- |
| ***Identification of cardiac arrest*** | | | | | | |
| *Call-handler continues questions after says no breathing* | *28* | *14.0%* | *Call-handler asks additional questions after established not breathing and unconscious* |  |  |  |
| *Caller uncertainty about breathing* | *141* | *70.5%* | *Answer about breathing is anything other than a definitive 'no'* |  | *1.000* |  |
|  |  |  |  |  |  |  |
| ***Defibrillator*** | | | | | | |
| Caller relaying instructions to fetch defibrillator | 10 | 5.0% | Caller giving instructions to fetch defibrillator to others on scene | ‘Can somebody go to [location] and get the defibrillator?' | 1.000 |  |
| Confusion about defibrillator | 29 | 14.5% | Request to get defibrillator adds delay due to not knowing what it is etc. | ‘If there’s what sorry?' |  | 0.589 |
|  |  |  |  |  |  |  |
| ***COVID*** | | | | | | |
| COVID screening questions | 14 | 7.0% | COVID screening questions and responses (*implemented by SAS during COVID pandemic*) |  | 0.643 |  |
| Instruction to cover mouth with cloth | 28 | 14.0% | Any talk related to instruction to cover mouth with cloth (*during COVID pandemic in 2020*) | ‘A towel over his face? Wait a sec' |  | 0.955 |
|  |  |  |  |  |  |  |
| ***Psychological / behavioural*** | | | | | | |
| Concern about doing harm | 17 | 8.5% | Caller expresses any concern about possible harm to patient | ‘I'm worried I'll bang her head' |  | 0.917 |
| Concern about ability to physically achieve | 66 | 33.0% | Caller expresses doubts that they can physically achieve (as opposed to discussing immovable objects etc) | ‘I’ve not got much strength cause I’m disabled' |  | 0.966 |
| Concern too late / futile | 35 | 17.5% | Caller expresses that they think it’s too late/ futile/ CPR won’t work/pointless | ‘Please he’s no gonnae make it' | 0.615 | 0.955 |
| Don't know how (to do CPR) | 16 | 8.0% | Caller expresses they don't know how to do CPR | ‘I don’t know what to do'. |  | 0.874 |
| Emotion | 91 | 45.5% | Any expressed emotion. Indicated emotion on transcript - bold, exclamation marks or in brackets e.g. [upset] | ‘[wailing] mum! ' | 0.828 | 0.726 |
| anger | 11 | 5.5% |  |  |  |  |
| panic | 27 | 13.5% |  |  |  |  |
| shock | 50 | 25% |  |  |  |  |
| upset | 61 | 30.5% |  |  |  |  |
| Rescuer wants to do something different from instruction | 28 | 14.0% | Caller/someone else on scene expresses desire to do something different from what call-handler is instructing | ‘I'm putting them in the recovery position’' | 0.483 | 0.587 |
| Trying to communicate seriousness of situation | 37 | 18.5% | Caller tries to communicate seriousness and urgency to CH (outside of responding to questions) | ‘We do really need someone out she is no looking good' |  | ~ |
|  |  |  |  |  |  |  |
| ***Other*** | | | | | | |
| Caller not with patient | 16 | 8.0% | Caller states they are not with patient (or it can be implied from what they say) | ‘I’ve came into another room to try not to, to stress her anymore' |  |  |
| Communication challenges | 96 | 48.0% | Difficulty communicating between caller and call-handler due to technical and situational factors | not being heard / having to put phone down to follow instructions / line distortion etc. | 0.800 | 0.670 |
| Clarifying questions | 51 | 25.5% | Caller asks clarifying questions | ‘I need to get her on the floor?’ | 0.318 | 0.725 |
| Multiple rescuers | 36 | 18.0% | Multiple people talk on call |  | 0.500 | 0.508 |
| Overdose related communication | 13 | 6.5% | Communication related to overdose situation | ‘He’s he’s took an overdose o’ heroin!’ | 1.000 |  |
| Providing (unrequested) medical information | 73 | 36.5% | Caller gives unprompted additional medical information (not asked by call-handler) | ‘His legs are cold' | 0.583 | 0.867 |
|  |  |  |  |  |  |  |
| Physical challenges | 77 | 38.5% | Any issues related to physically moving patient (n.b. expressed concerns about physical capability listed under psychological/behavioural, above) |  | 1.000 |  |
| Too heavy | 25 | 12.5% |  | ‘But you’ve got to understand this man is about 20 stone' |  |  |
| Awkward position | 59 | 29.5% |  | ‘He’s stuck between a bed and a wardrobe' |  |  |
| Situational | 15 | 7.5% | Particular things about situation that might delay CPR | ‘Right but the problem is that the key has been lost for the front door so you’ll need to come to the back window'. |  |  |
